# Editorial: Cooperation of MSC and biomaterials for cell expansion and tissue engineering

**DOI:** 10.3389/fbioe.2024.1440925

**Published:** 2024-06-17

**Authors:** Yan Li

**Affiliations:** ^1^ School of Biomedical Engineering, Shenzhen Campus of Sun Yat-sen University, Shenzhen, China; ^2^ Guangdong Provincial Key Laboratory of Sensor Technology and Biomedical Instrument, Sun Yat-sen University, Guangzhou, China

**Keywords:** mechanosensing, 3D cell carriers, wound healing, bone regeneration, MSC

## Introduction

Mesenchymal stem cells (MSCs) are a type of multipotent stem cells derived from the mesoderm, possessing a high capacity for self-renewal and differentiation into various cell types. They can be easily isolated from sources such as adipose tissue, bone marrow, and umbilical cord. MSCs exert beneficial functions in anti-inflammatory responses, tissue repair, and cellular homeostasis through paracrine secretion of various cytokines, interacting with immune cells and other cell types. These excellent characteristics make them ideal for tissue engineering applications.

Two-dimensional (2D) microenvironment often led to the loss of stem cell properties of MSCs, with decreased proliferation and differentiation capacities with prolonged culture duration. Numerous studies have demonstrated that biomaterials well maintained the vitality of MSCs, providing more tissue-like structures, and simulated the *in vivo* microenvironment, thereby facilitating cell-cell and cell-extracellular matrix interactions. This promoted the *in vitro* adhesion, migration, proliferation, and multipotent potential of MSCs, as well as the expression of bioactive factors.

In recent years, interdisciplinary research efforts have focused on leveraging the synergistic interplay between MSCs and biomaterials to achieve enhanced outcomes in cell expansion and tissue engineering applications.

## Dynamic mechanosensing in MSCs osteogenesis: unveiling the role of YAP

The mechanotransduction pathways governing MSCs behaviors, particularly in the context of osteogenesis, have emerged as critical determinants of tissue regeneration. Studies, such as the one by Kim et al., elucidated the pivotal role of yes-associated protein (YAP) in translating mechanical cues into cellular responses, thereby modulating MSCs osteogenic differentiation. Employing cyclic mechanical stretching demonstrated the dynamic nature of YAP mechanosensing, offering valuable insights into the regulation of MSCs fate by mechanical stimuli. These findings underscore the significance of dynamic mechanobiology in optimizing strategies for bone tissue engineering.

## Three-dimensional (3D) cell carriers for MSCs Cultivation

Traditional 2D culture systems often fail to recapitulate the complex microenvironment essential for maintaining MSCs stemness. Innovative approaches utilizing 3D cell carriers, such as those incorporating decellularized extracellular matrix (dECM) components, have emerged as promising platforms for enhancing MSC functionality. For instance, Li et al. established a 3D cell carrier based on PET microfibers interpenetrated with pulmonary decellularized extracellular matrix. Such composite carriers exhibited significant performance in promoting the adhesion, proliferation, and paracrine functions of MSCs.

## Precision modeling of bone reconstruction via 3D bioprinting

The advent of 3D bioprinting has revolutionized the field of tissue engineering by precisely fabricating complex tissue constructs with spatial control over cellular organizations and biomaterial compositions. Lv et al. utilized 3D bioprinting to develop a bone reconstruction model. Precursor cells of osteoblasts and osteoclasts were integrated to analyze the roles of scaffold in bone remodeling. The results demonstrated that the scaffold effectively promoted cell adhesion and growth, exhibiting a favorable effect on inducing differentiation. This provided unparalleled opportunities for modeling bone remodeling processes *ex vivo*. These models not only offered important insights into the mechanisms of bone regeneration but also served as valuable platforms for evaluating novel therapeutic interventions.

## Biomaterials facilitating wound healing and skin regeneration

The development of advanced wound dressings capable of modulating the wound microenvironment and promoting tissue regeneration represents a frontier in wound care research. Utilizing a combination of antimicrobial peptides (AMPs) and bioactive components, such as hydroxyapatite (HAp) and silk fibroin (SF), Chen et al. engineered smart wound dressings with multifunctional properties. These dressings not only exhibited potent antimicrobial activity but also facilitated cell proliferation, collagen deposition, and angiogenesis, thereby accelerating the healing of infected wounds while minimizing scar formation.

dECM derived from adipose-derived stem cells (ADSCs) holds immense potential for promoting skin regeneration and remodeling. The research conducted by Zhang et al. showed that ADSC-dECM, incorporated into biomaterial-based patches, enhanced cell proliferation, migration, and tissue regeneration both *in vitro* and *in vivo*. By harnessing the regenerative properties of ADSC-dECM, the approach offered a new strategy for addressing the unmet clinical needs in skin tissue engineering and wound healing.

## Targeted recruitment of endogenous stem cells for periodontal regeneration

Effective regeneration of periodontal tissues hinges on the selective recruitment of endogenous progenitor cells with regenerative potential to the defect site. The strategy utilizing antibody-conjugated microspheres, as demonstrated by Zou et al., enabled precise targeting of specific cell populations, such as periodontal ligament cells (PDLCs), to promote *in situ* bone regeneration. By harnessing the inherent regenerative capacity of endogenous stem cells, the approach provided a promising avenue for achieving guided tissue regeneration in periodontal therapy.

## Conclusion

In summary, the convergence of MSCs biology, biomaterials science, and tissue engineering has paved the way for transformative advancements in regenerative medicine. By combining the unique characteristics of MSCs and biomaterials, researchers continue to innovate and develop next-generation therapeutic approaches to achieve large-scale cell expansions, effective stem cell delivery, and disease treatment. This collaborative efforts hold the potential to alter clinical practice and improve patient outcomes in tissue repair and regeneration.

